# Sexual dysfunction in women with genital warts: a cross-sectional study

**DOI:** 10.1186/s12905-025-03691-6

**Published:** 2025-04-16

**Authors:** Mohamed S. Zaky, Zakaria M. Obaid, Ahmed M. Youssef, Asmaa D. Elmenawy, Mohamed L. Elsaie

**Affiliations:** 1 Department of Dermatology, Venereology and Andrology, Damietta Faculty of Medicine, Al-Azhar University, Damietta, Egypt; 2 Department of Public Health and Community Medicine, Damietta Faculty of Medicine, Al-Azhar University, Damietta, Egypt; 3 Department of Dermatology, Venereology and Andrology, Medical Research and Clinical Studies Institute, National Research Centre, Giza, Egypt

**Keywords:** Sexual Dysfunction, HPV, Genital warts, DLQI

## Abstract

**Purpose:**

This study aimed to evaluate sexual dysfunction and quality of life in women with genital warts.

**Methods:**

A total of 100 women were included if they were aged 18–45 years, had genital warts (GWs) for at least three months, and were in a stable marital relationship for at least six months. All participants completed the Female Sexual Function Index (FSFI) and the Dermatology Life Quality Index (DLQI) questionnaires.

**Results:**

Showed that the lowest mean scores were observed in the domains of orgasm (1.91 ± 1.07), arousal (2.82 ± 1.12), satisfaction (3.01 ± 1.01), pain (3.11 ± 1.34), desire (3.37 ± 1.01), and lubrication (3.64 ± 1.32). Total FSFI score was 17.64 ± 6.15. The presence of GWs had a significant very large impact on patients QOL in 49% of participants (*p* < 0.001).

**Conclusions:**

Results demonstrated sexual and quality of life affection among females complaining of genital warts. Sexual health and dysfunction should be routinely assessed in women presenting with genital warts.

## Introduction

Genital warts, caused by human papillomavirus (HPV), have a global annual prevalence of 160–289 per 100,000 individuals, with peak incidence occurring at ages 25–29 years in men and 20–24 years in women [1].

A number of factors such as age, gender, level of education, age of sexual contact as well as sexual habits have been confirmed to affect the nature and incidence of contracting genital warts [2]. According to some studies, HPV and GWs affects patients physically and psychologically. Initial reactions of patients include anger, depression, isolation, shame, and guilt [3].

Female sexual dysfunctions (FSD) include dyspareunia, female orgasmic disorder, female sexual arousal disorder, vaginismus, and sexual difficulties with an estimate of 40% of female population experiencing FSD [3]. Besides anxiety, pain, discomfort and depression; GWs can alter people's sexual desire and threaten their sexual health. GWs may affect sexual life, self-image, self-esteem, emotions, daily activities, and the quality of life, because of pain and discomfort, anxiety, and depression [4].

Factors contributing to decreased sexual desire, arousal, and orgasm in women with genital warts include concerns about lesion recurrence, fear of transmission, anxiety about spousal infidelity, misconceptions about HPV or general sexual education, and psychological distress following diagnosis [5]. This study was designed to evaluate sexual dysfunction and quality of life in females with genital warts.

## Methods

This cross-sectional study was approved by the local ethics committee and the institutional review board (IRB). Confidentiality was maintained throughout the study, and collected data were used solely for research purpose.

### History taking

100 Females were determined eligible for inclusion if they were between 18–45 years, complaining of GWs for at least 3 months and had been in a stable marital relationship for the past 6 months. Pregnant females, those with history of mastectomy, oopherectomy, ongoing sexual disorders and those with history of genital mutilation and taking psychotropic drugs were excluded from the study.

### Filling out the Female Sexual Function Index (FSFI) questionnaire

All women were invited to attend a personal face-to-face interview done by a female investigator. The structured interviews were based on the19-item FSFI questionnaire [5] which was translated, validated and reproduced into Arabic [6].

FSFI assesses sexual functioning in women in six separate domains (desire, arousal, lubrication, orgasm, satisfaction, pain). A total score of 26.5 was considered as the optimal cut off point to distinguish between women with SD and those with normal sexual function.

### Assessment of quality of life

The impact of genital warts on QOL was assessed using Dermatology Life Quality Index (DLQI) questionnaire designed for use in patients over the age of 16 [7] which was translated into Arabic and tested for validity and reproducibility [8].

### Statistical analysis

The statistical analysis was carried out using statistical package for social science (SPSS) version 22 (SPSS Inc., Chicago, Illinois, USA). Qualitative data were presented as number and percent, Quantitative data were tested for normality by Kolmogrov-Smironv test then described as mean and standard deviation for normally distributed data and median and range for non normally distributed. The appropriate statistical test was applied according to data type with the following suggested tests: Chi-Square for categorical variable, Student t test and Mann Whitney U test. Sample size calculation was based on prevalence of sexual dysfunction among cases with genital warts retrieved from previous research [9]. Using Epi info version 7.2.4.0 to calculate sample size based on 83% expected prevalence, 95% CL with acceptable margin of error = 5; a sample size of 100 was determined to be adequate for statistical analysis.

## Results

The mean age of participants was 32.5 ± 7.5 years, with a mean disease duration of 20.03 ± 10.39 months and a mean marriage duration of 4.87 ± 2.48 years. The majority (52%) of participants were living in rural areas and 42% had basic education (ability to read and write) while 28% were high school graduates and 14% had a university degree. While 32% of participating women were employed, only 12% had knowledge about HPV. The majority (74%) complained of multiple GWs and 42% developed recurrent GWs. Seventy-eight (78%) reported their husbands to be infected with GWs and only 18% used condoms Tables 1 and 2.

**Table 1:** Distribution of demographic data in the studied group

Variables	Studied group *N* = 100
Age (years) Mean ± SD	32.5 ± 7.5
Duration of marriage (years)	4.87 ± 2.38
Disease duration (month)	20.03 ± 10.39

**Table 2:** Distribution of socio-demographic results in the studied group

**Variable**	**Studied group** *N* = 100
Residence	
Urban	48 (48%)
Rural	52 (52%)
Patient education	
Not educated	16 (16%)
Basic (read and write)	42 (42%)
Secondary	28 (28%)
University	14 (14%)
Working status	
Working	32 (32%)
Not working	68 (68%)
HPV knowledge	
Some	12 (12%)
Little	32 (32%)
Non	56 (56%)

*HPV* Human papillomavirus

The FSFI includes 19 items optimally illustrating the 6 dimensions. The summary score ranges from 2 to 36, with low scores indicating more severe FSD. The sexual function domains showed the lowest mean scores to be related to orgasm (1.91 ± 1.07), arousal (2.82 ± 1.12), satisfaction (3.01 ± 1.01), pain (3.11 ± 1.34), desire (3.37 ± 1.01,), and lubrication (3.64 ± 1.32). Total FSFI score was 17.64 ± 6.15. The presence of GWs had a significantly large impact on patients QOL in 49% of participants (*p* < 0.001). The domains with the greatest impact on the patients'quality of life were embarrassment, problems with partner, friends or relatives, as well as sexual difficulties Tables 3, 4, 5, 6 and 7.

**Table 3:** Interpretation of DLQI among the studied females

DLQI	Studied group *N* = 100	test	*P* value
Small effect on patient's life (2–5)	**5 (5%)**	**X**^**2**^** = 54.3**	** < 0.001**
Moderate effect on patient's life (6–10)	**46 (46%)**		
Very large effect on patient's life (11–20)	**49 (49%)**		

*SD* standard deviation; *DLQI* Dermatology Life Quality Index

*P* value > 0.05: Not significant, *P* value < 0.05 is statistically significant, p < 0.001 is highly significant

**Table 4:** FSFI scores among the patients (*n* = 100)

**FSFI**	**Mean ± SD**	**Range**
Desire domain	3.37 ± 1.01	1.4–5.64
Arousal domain	2.82 ± 1.12	0–5.1
Lubrication domain	3.64 ± 1.32	0–5.9
Orgasm domain	1.91 ± 1.07	0–4.4
Satisfaction domain	3.01 ± 1.01	1.4–5.4
Pain domain	3.11 ± 1.34	0–5.4
Total score	17.64 ± 6.15	3.8–27.9

*FSFI* Female Sexual Function Index

**Table 5:** Correlation between FSFI, DLQI and the studied variables

	**FSFI**		**DLQI**	
	**r**	**p**	**r**	**p**
**Age**	− 0.434	* 0.023	− 0.098	0.6
**Duration of marriage**	− 0.389	* 0.009	− 0.198	0.21
**Disease duration**	− 0.663	* < 0.001	− 0.614	* < 0.001

*DLQI* Dermatology Life Quality Index; *FSFI* Female Sexual Function Index

^*^ Significant, *P* value < 0.05 is statistically significant, *p* < 0.001 is highly significant

**Table 6:** Relation between FSFI; QOL and socio-demographic results

	**FSFI**	*P*** value**	**QOL**	*P*** value**
Residence				
Urban	16.9 ± 7.43	0.06	12.9 ± 4.7	< 0.001*
Rural	18.5 ± 4.65		9.05 ± 2.09	
Patient education				
None	16.7 ± 6.23	0.631	9.8 ± 4.3	0.288
Basic	16.5 ± 5.64		10.3 ± 2.91	
Secondary	18.3 ± 6.56		11.4 ± 3.46	
University	18.0 ± 7.23		11.6 ± 3.69	
Working status				
Working	17.3 ± 6.89	0.94	12.1 ± 2.97	0.007*
No work	17.4 ± 6.03		10.2 ± 3.34	
HPV knowledge				
Some	22.8 ± 1.85	* < 0.001	9.8 ± 4.17	0.39
Little	14.9 ± 6.23		11.3 ± 2.99	
Non	17.6 ± 5.41		10.6 ± 3.42	

*SD* standard deviation; *QOL* quality of life; *HPV* Human papillomavirus; *FSFI* Female Sexual Function Index

^*^ Significant, *P* value < 0.05 is statistically significant, *p* < 0.001 is highly significant

**Table 7:** Relation between FSFI; QOL and genital wart characteristics

	**FSFI**	*P*** value**	**QOL**	*P*** value**
No. of GW				
Single	21.6 ± 2.83	* < 0.001	7.89 ± 2.91	* < 0.001
Multiple	14.97 ± 6.92		10.82 ± 3.63	
Recurrence of GW				
Yes	13.1 ± 5.13	* < 0.001	10.78 ± 2.96	* 0.005
No	20.01 ± 4.82		8.84 ± 3.59	
Husband infection with GWs				
Yes	17.2 ± 6.31	0.84	10.7 ± 3.5	0.71
No	17.5 ± 6.12		10.4 ± 2.9	
Condom use				
Yes	18.7 ± 7.3	0.056	8.78 ± 2.1	0.29
No	15.6 ± 5.9		9.75 ± 3.8	
Treatment type				
Cryotherapy	16.5 ± 6.3	0.72	10.2 ± 3.25	0.11
Podophyllin solution	16.6 ± 6.7		8.3 ± 3.3	
Candida antigen	19.1 ± 3.4		8.03 ± 2.6	

*SD* standard deviation; *QOL* quality of life; *HPV* Human papillomavirus; *FSFI* Female Sexual Function Index; *GW* Genital wart

^*^Significant, *P* value < 0.05 is statistically significant, *p* < 0.001 is highly significant

Factors associated with FSD showed significant direct correlations (*P* -value < 0.05) between female having sexual dysfunction and women age (*p* = 0.023), duration of marriage (*p* = 0.009), disease duration (*p* = 0.001), HPV knowledge (*p* < 0.001), GWs (number, recurrence; *P* < 0.001). There were non-significant correlations (*P*-value > 0.05) between participants having sexual dysfunction and residence (*P* = 0.06), level of education (*P* = 0.63), working status (*P* = 0.94), husband infection with GWs (*P* = 0.84), condom use (*P* = 0.06), and type of treatment (*P* = 0.72). Quality of life was significantly higher among participants living in urban areas (*p* < 0.001) and those who were employed (*p* = 0.007).

## Discussion

In current study, there was significant correlation between FSFI, age, duration of marriage and disease duration while there was significant correlation between DLQI and disease duration in the studied group. Most studies investigating demographic and social factors in women with GWs have identified duration of marriage, age, education level, residence, and HPV knowledge as key factors affecting sexual function [10].

In the present study GWs were found to affect all dimensions of sexual function in females. A higher prevalence of sexual dysfunction was found in women complaining of genital warts that significantly affected their QOL. Findings from different studies remain contradictory regarding the impact of genital warts on sexual dysfunction in females. Our results were consistent with a number of previous studies [11–15].

Sexual dysfunction complaints were inversely related to women's level of education and income. Also, size of the warts, number of treatments, and the duration of treatment were inversely related to sexual dysfunction [16]. One study reported a 68% reduction in libido and a 42% decrease in sexual intercourse frequency, with 19% of participants experiencing partner rejection and 71% unable to enter a new relationship [17]. Half of the participants in another study on Taiwanese women with high risk of HPV had problems in their sexual life, the most important of which was a decrease in the sexual desire and frequency of intercourse [18].

A number of studies indicated that women experienced a decline in sexual functioning related to desire, arousal, orgasm, pain, lubrication, as well as satisfaction among which diminished libido was most prominent [11, 12, 15, 16]. The occurrence of sexual dysfunction in women was directly correlated with their age, length of marriage, duration of the disease, knowledge about HPV, number of warts, and recurrence of warts. In contrast, there were insignificant associations between the level of education, place of residence, occupation, spouse's infection with GW, condom usage, and type of treatment, and female sexual dysfunction [11, 12].

In contrast to these findings, a cross-sectional study on 100 refugees living in Turkey and complaining of GWs; no effect on their sexual function was reported. Discrepancies in results were attributed to lack of knowledge about HPV among the study group (refugees) and the lower perceived significance of GWs for them [14]. Higher education and location of warts on the clitoris of females were among factors that negatively affected Colombian women's sexual lives as per a cross sectional designed study [4].

Almost all female participants reported a significant negative impact on their quality of life with 50% reporting very much impact of GWS on their life quality. The domains with the greatest impact on the patients'quality of life were embarrassment, problems with partner, friends or relatives, sexual difficulties. The majority of studies reported women complaining of GWs to experience significant psychosocial and psychosexual impacts on their lives compared to the general population [19–21]. Women and men with GWs reported moderate to severe negative psychosocial and psychosexual impacts but GWs'negative impacts are more common in women than in men [4, 20, 21].

The major limitation of our research was the absence of a control group, limiting the ability to assess the direct impact of GWs, a relatively small sample size, and the selection of patients from a single institution, which may contribute to selection bias. Given the sensitive and delicate nature of sexual health and the potential for cultural differences in sexual behaviour, another limitation could have been the reluctance to communicate and report about sexual subjects.

## Conclusion

In conclusion, a significant association was observed between genital warts and lower FSFI scores in women. Sexual health and dysfunction and their impact on quality of life should be part of the integral routine investigation for females complaining of genital warts. Further research is recommended in diverse populations.

## Data Availability

The data that support the findings of this study are available from the corresponding author upon reasonable request.

## Abbreviations

DSM: Diagnostic and statistical manual of mental disorders
FSFI: Female sexual functioning index
GWs: Genital warts
HPV: Human Papillomavirus
FSD: Female sexual dysfunction
DLQI: Dermatology Life Quality Index
QOL: Quality of life
